# The Pharmacological and Clinical Roles of Antiemetics: A Narrative Review

**DOI:** 10.7759/cureus.77370

**Published:** 2025-01-13

**Authors:** Alan D Kaye, Donovan B Turpin, Shivam Shah, Brennan Abbott, Alex V Hollander, Caroline R Burroughs, Sarah H Myers, Shahab Ahmadzadeh, Jibin Mathew, Adam M Kaye, Sahar Shekoohi, Giustino Varrassi

**Affiliations:** 1 Department of Anesthesiology, Louisiana State University Health Sciences Center, Shreveport, USA; 2 School of Medicine, Louisiana State University Health Sciences Center, Shreveport, USA; 3 Department of Internal Medicine, Louisiana State University Health Sciences Center, Shreveport, USA; 4 Department of Pharmacy Practice, Thomas J. Long School of Pharmacy and Health Sciences, University of the Pacific, Stockton, USA; 5 Department of Pain Medicine, Fondazione Paolo Procacci, Rome, ITA

**Keywords:** antiemetics, chemotherapy, gastroenteritis, motion sickness, nausea, vomiting

## Abstract

Emesis (e.g., vomiting and nausea) can be a nebulous condition to treat, as it has many and highly varied etiologies. Understanding the underlying causes of emesis is crucial in selecting the appropriate antiemetic therapy. At present, there are a plethora of medications on the market to treat emesis, known as antiemetics. For the healthcare professionals managing emesis, key concerns include efficacy, the onset of effects, and potential adverse actions of these drugs. The present investigation reviews different classes of antiemetics and specific clinical uses for each based on pharmacological action. In addition, a few emerging treatment options are discussed, including cannabinoids, vitamin B6 (pyridoxine), and combination formulations such as Diclegis (doxylamine-pyridoxine), prompting avenues for future roles in the management of emesis.

## Introduction and background

Emesis is a physiological response to many triggers [1]. The body forcefully expels stomach contents through the mouth as a defense mechanism against potentially harmful substances or in reaction to other systemic imbalances. With the loss of these fluids, common secondary issues include dehydration with hypovolemia, electrolyte imbalance, and acid-base disorders [2]. As a result, treatment for emesis almost ubiquitously includes aggressive fluid and electrolyte replacement to avoid adverse effects, especially hyponatremic arrhythmias [3]. Emesis is often precipitated by nausea, a common symptom and side effect in many clinical scenarios. There is a wide variety of drugs that may be used to suppress an emetic response or the nausea that initiated it; they are broadly termed "antiemetics."

With an innumerable number of causes of emesis, there are just as many different mechanisms of action for antiemetic drugs. Among the more commonly used are antihistamines, such as diphenhydramine, dimenhydrinate, meclizine, and promethazine; these drugs block H1 and H2 histamine receptors. There are also H2 receptor antagonists (H2RAs), which, due to their specificity, show reduced side effects. Ondansetron, granisetron, and other drugs of similar structure block 5-hydroxytryptamine-3 (5-HT3) serotonin receptors. Among other drugs, benzamides such as metoclopramide exert their action by blocking D2 dopamine receptors. Less commonly prescribed antiemetics include other D2 receptor blockers, such as phenothiazines, for example, prochlorperazine. Dexamethasone, a glucocorticoid, has also been shown to have antiemetic properties. Aprepitant (or its prodrug, fosaprepitant) exerts its effect by blocking neurokinin-1 (NK-1) neurokinin receptors [4]. Benzodiazepines, mainly used as central nervous system (CNS) depressants, act by enhancing the activity of gamma-aminobutyric acid (GABA) receptors, which has demonstrated some efficacy in decreasing anticipatory nausea and subsequent emesis [5].

In the present investigation, therefore, we evaluate strategies for choosing a specific medication based on its pharmacodynamic and pharmacokinetic considerations; our aim is to compile the swaths of current literature into a concise and accessible review resource. Furthermore, we address possible side effects and contraindications in which a drug should be avoided or discontinued. Finally, we briefly examine a few new or controversial drugs that are beginning to be used in trials and clinics for antiemetic purposes.

## Review

Emesis etiologies and choosing a drug

Understanding the underlying causes of emesis is crucial in selecting the appropriate antiemetic therapy because different etiologies engage specific pathways in the body responsive to various classes of medications [6]. The most common emesis triggers arise from gastrointestinal (GI) tract disturbances, including infectious gastroenteritis, gastritis, and peptic ulcer disease [7]. In these cases, the irritation of the stomach lining stimulates the vagal and splanchnic nerves, which signal the vomiting center in the brain. Diseases causing obstruction, such as ileus or bowel obstruction, can also induce vomiting [8]. Central nervous system conditions such as migraines, head trauma, intracranial pressure, and brain tumors often trigger emesis [9]. Several pharmacological agents are known to cause emesis, most notably chemotherapeutic drugs, opioid analgesics, and anesthetics [10]. Chemotherapy-induced nausea and vomiting (CINV) is one of the most studied forms of drug-induced emesis and is mediated through the release of serotonin (5-HT3) from the enterochromaffin cells in the gut [11].

Dopaminergic drugs, nonsteroidal anti-inflammatory drugs (NSAIDs), and certain antibiotics also contribute to nausea and vomiting [10]. These disorders affect the chemoreceptor trigger zone (CTZ), located in the area postrema of the brainstem, which is particularly sensitive to changes in blood chemistry or to direct central insults [12]. Electrolyte imbalances or disturbances in acid-base balance also play a significant role in triggering vomiting [13]. Emotional stress, anxiety, and even the anticipation of stressful events (such as chemotherapy) can stimulate emesis [14]. These instances are usually mediated by higher cortical centers interacting with the vomiting center in the medulla. Nausea and vomiting during pregnancy (NVP), especially in the first trimester, is a well-known phenomenon that affects about 70%-90% of pregnant women [15,16]. Hyperemesis gravidarum, a severe form of NVP, can lead to significant malnutrition if not managed promptly [17]. Among women with hyperemesis gravidarum, it is estimated that the fetal loss rate is about 37%, and it is also associated with higher rates of maternal death [18,19]. The etiology is believed to be multifactorial, involving hormonal changes (e.g., elevated levels of human chorionic gonadotropin {hCG} and growth differentiation factor 15 {GDF15}) and possibly a hypersensitivity to estrogen [20-22]. The vestibular system, located in the inner ear, is responsible for balance. Disruptions to this system, such as in cases of motion sickness or Meniere's disease, activate histamine (H1) and muscarinic receptors in the vomiting center [23].

The choice of antiemetic should be based on the etiology of the nausea and vomiting, as different mechanisms are responsible for triggering the emetic response in each case. A tailored approach targeting the appropriate pathway increases efficacy. 5-HT3 receptor antagonist drugs, such as ondansetron and granisetron, are effective in managing nausea related to chemotherapy, radiation, and postoperative states [24]. Blocking serotonin receptors in both the central and peripheral nervous system helps alleviate CINV, where serotonin is released from the gut lining and stimulates the vagus nerve. Dopamine antagonist medications such as metoclopramide and domperidone work by inhibiting dopamine receptors in the CTZ [25]. They are effective in treating nausea related to GI disturbances, metabolic imbalances, and drug-induced emesis. Antihistamines and anticholinergics are used for nausea due to motion sickness or vestibular disturbances [26]. Drugs such as diphenhydramine (H1 antagonist) and scopolamine (muscarinic antagonist) are preferred. These drugs reduce the excitability of the vestibular system and prevent the activation of the vomiting center [27]. Aprepitant is an example of a drug that blocks neurokinin-1 (NK-1) receptors and is used in conjunction with 5-HT3 antagonists and corticosteroids to prevent delayed CINV [28]. Dexamethasone, a corticosteroid, is commonly used as an adjunctive antiemetic in the setting of chemotherapy and postoperative nausea related to anti-inflammatory properties, which likely reduce the release of pro-emetic cytokines [29-31]. For anticipatory nausea, often seen in chemotherapy patients, benzodiazepines such as lorazepam are effective due to their anxiolytic and sedative properties, addressing both the psychological and physical aspects of emesis [32].

Drug delivery and metabolism

In the management of nausea and vomiting, the route of drug delivery can significantly influence treatment efficacy, particularly in patients who are actively vomiting and cannot tolerate oral medications. Therefore, alternative methods of drug administration play a critical role in clinical settings. There are six routes of drug delivery: oral, intravenous (IV), intramuscular (IM), rectal, transdermal, and sublingual/buccal [33].

Oral formulations are the most common delivery method but are unsuitable in cases where patients are actively vomiting or have GI obstructions [34]. However, they are ideal for mild cases of nausea and long-term maintenance [35]. Ondansetron is available in both oral and orally disintegrating tablets (ODT), making it easier for patients to take during milder nausea [34]. IV administration provides the fastest onset of action, particularly for severe or acute emesis, because the medication's bioavailability is directly linked to the circulatory system and is quickly delivered to the site of action [36]. Ondansetron and metoclopramide are frequently administered this way in clinical settings [34]. IM injections offer an alternative for patients who cannot tolerate oral medications and where IV access is not readily available [37]. IM delivery can be painful, may result in unpredictable drug absorption, and carries the risk of sterile abscess formation or tissue fibrosis [38]. Prochlorperazine, haloperidol, and metoclopramide are a few examples of antiemetics often administered via IM injection in emergency settings [39]. Rectal suppositories can be helpful for drugs with poor oral stability, solubility, or permeability and for patients unable to take medication orally due to nausea, unconsciousness, or swallowing difficulties [40]. Drugs such as promethazine, ondansetron, and prochlorperazine are often administered rectally for rapid absorption in these cases. Scopolamine is available as a transdermal patch, making it a convenient option for patients prone to motion sickness or those requiring continuous low-dose antiemetic coverage, such as during long trips or postoperative recovery [41]. Similarly, granisetron is available through a transdermal patch (Sancuso®) and, as such, is the only patch approved to treat CINV. Its label recommends application up to 48 hours before therapy and removal no less than 24 hours afterward [42]. Ondansetron is available in an orally disintegrating tablet (ODT) form, which dissolves under the tongue or on the buccal mucosa. The ODT is absorbed through the gastrointestinal tract when saliva is swallowed, and it has absorption kinetics similar to those of the conventional ondansetron tablet [43]. This makes it an effective option for patients who are experiencing mild-to-moderate nausea without continuous vomiting.

Once administered, antiemetic drugs undergo metabolism primarily in the liver, where they are broken down by cytochrome P450 (CYP) enzymes [44]. Variations in these enzymes between individuals can impact the metabolism and effectiveness of drugs, necessitating personalized dosing in some cases. Ondansetron is metabolized by multiple enzymes, including CYP3A4, CYP1A2, and CYP2D6 [45]. Genetic polymorphisms in these enzymes can lead to variability in the drug's efficacy and side effect profile [46]. For instance, patients with poor CYP2D6 metabolism may experience prolonged drug action and increased risk of side effects [47]. Metoclopramide undergoes metabolism primarily via CYP2D6, and thus, poor metabolizers may experience exaggerated effects of the drug, leading to an enhanced risk of side effects such as extrapyramidal symptoms [48]. Scopolamine is metabolized in the liver and excreted primarily in the urine. The slow-release mechanism of the transdermal patch ensures consistent plasma levels over a prolonged period, making it ideal for preventing motion sickness and postoperative nausea over 72 hours [49].

Cases with liver or kidney dysfunction may require dose adjustments due to altered metabolism and the excretion of antiemetics. In patients with liver disease, drugs metabolized via hepatic enzymes, such as ondansetron, may accumulate to toxic levels, leading to adverse effects [50]. Similarly, in patients with renal impairment, drugs that are primarily excreted by the kidneys may require careful monitoring and dose adjustment [51].

Side effects and contraindications

Antiemetic medications are effective in managing nausea and vomiting; however, each class comes with a range of potential side effects and contraindications that should be considered when prescribing. First-generation antihistamines, such as diphenhydramine, meclizine, and promethazine, easily cross the blood-brain barrier and cause noticeable side effects such as sedation, confusion, dry mouth, and QT prolongation [52]. The sedative side effects are especially concerning in elderly patients, who are already at a higher risk of cognitive impairment [52]. However, H2RAs such as famotidine, cimetidine, and nizatidine do not easily cross the blood-brain barrier, resulting in fewer side effects than first-generation options. Side effects such as diarrhea, constipation, fatigue, and confusion have been reported but are uncommon [52]. One notable exception is cimetidine. It has been shown to cause gynecomastia, impotence, and galactorrhea due to its anti-androgenic effects. These anti-androgenic effects are not seen in other H2RAs [53]. Antihistamines are relatively contraindicated in patients with QT prolongation or those using drugs that may prolong the QT interval. Other contraindications include urinary retention, liver disease, and glaucoma [52].

The most common side effects of selective serotonin receptor (5-HT3) antagonists in CINV therapy include headache, fatigue, and constipation [54]. They can also cause a dose-dependent QT prolongation, which is generally insignificant in healthy patients [54]. The use of 5-HT3 receptor antagonists for nausea from apomorphine therapy is contraindicated, as it can cause decreased consciousness and hypotension; trimethobenzamide, a phenothiazine, should be used as the alternative antiemetic therapy in this case [55,56]. The box labeling for 5-HT3 receptor antagonists warns of the risk of serotonin syndrome when these drugs are combined with other serotonergic drugs, but the evidence of this is limited [57].

Short-term dexamethasone use for CINV has mild side effects, including insomnia and mood changes, and can cause hyperglycemia in some cases [58,59]. Glucocorticoids are relatively contraindicated in patients with diabetes mellitus or active infections due to their hyperglycemic and immunosuppressive risks [59].

Dopamine antagonists are associated with extrapyramidal symptoms such as dystonia, akathisia, and tardive dyskinesia [59,60]. These side effects are especially prominent with long-term use, and the box warning for metoclopramide strongly discourages treatment for longer than 12 weeks. They should be prescribed with caution to elderly patients and children as they are more prone to developing extrapyramidal symptoms. Dopamine antagonists are contraindicated in cases with Parkinson's disease due to the risk of worsening motor symptoms. Anticholinergic drugs, most commonly scopolamine, are used to treat motion sickness or prophylactically perioperatively. Side effects are generally mild, including dry mouth, constipation, and blurred vision [61]. These drugs are contraindicated in patients with glaucoma or those at risk for urinary retention due to their anticholinergic effects [59,61]. Finally, NK-1 receptor antagonists, such as aprepitant and fosaprepitant, prevent the release of substance P, an inducer of vomiting [4]. They are generally well-tolerated but may cause mild side effects such as fatigue and dizziness [62]. There have also been case reports of hypersensitivity reactions such as anaphylaxis and anaphylactic shock [59,62]. These drugs also inhibit CYP3A4, potentially causing drug-drug interactions with other medications metabolized through this pathway [4].

Emerging or unique treatment options

More recently, alternative treatments for nausea and vomiting have gained attention for patients who do not respond well to traditional antiemetics. These therapies include cannabinoids, vitamin B6 (pyridoxine), and combination formulations such as Diclegis (doxylamine-pyridoxine). In addition, droperidol is re-emerging in the United States for its antiemetic properties. Although they are less widely prescribed or studied than traditional antiemetics, these therapies have shown promise.

Cannabinoids such as tetrahydrocannabinol (THC) and cannabidiol (CBD) have been explored for their antiemetic properties, especially in patients undergoing chemotherapy. Dronabinol and nabilone are both synthetic THCs Food and Drug Administration (FDA)-approved to treat chemotherapy-induced nausea after the failure of first-line antiemetics [63]. Epidiolex is a synthetic CBD that is FDA-approved to treat two rare seizure disorders but has no indication for its use as an antiemetic [63,64]. Cannabinoids' use as antiemetics is often limited by psychoactive effects such as euphoria, anxiety, and paranoia [63]. Cannabinoid formulas containing only CBD have less potential for negative psychoactive effects compared to THC-based formulas.

Vitamin B6 (pyridoxine) in combination with doxylamine has been used as first-line treatment for nausea and vomiting during pregnancy (NVP). Diclegis, which combines doxylamine succinate with pyridoxine hydrochloride, is FDA-approved for the treatment of NVP [65]. Common side effects of this combination drug include drowsiness and dry mouth, with a minimal risk of serious side effects [65]. It is contraindicated with monoamine oxidase inhibitors, which exacerbate the CNS and anticholinergic effects [65]. Droperidol is an antipsychotic whose label contains a "black box" warning of increased risk for cardiac complications such as QT prolongation; however, it was reintroduced in the United States in 2019 by American Regent, Inc. with off-label use as a cost-effective antiemetic [66]. The FDA's original decision to apply the label in 2001 was based on data from doses up to two orders of magnitude greater than the antiemetic dosing recommendations, and it is unclear whether there are still significant cardiac risks at these low doses [66]. There seem to be no clear differences in side effects compared to 5-HT3 receptor antagonists, which are also known to prolong the QT interval [67]. Further research should be conducted to assess droperidol's safety at lower dosages. Still, it should not be used with conditions or drugs that cause prolonged QT interval.

In addition to those mentioned above, several novel therapies are being investigated for their antiemetic properties. For instance, ghrelin receptor agonists are being studied as potential nausea treatments, though the evidence is minimal. Ghrelin's role in appetite stimulation and gastric motility may relieve nausea by enhancing gastric emptying [68]. Another emerging option involves central neuromodulators such as tricyclic antidepressants, gabapentin, or olanzapine, an atypical antipsychotic that has shown promise in treating chronic nausea resembling neuropathic pain pathways [69-71]. Olanzapine is an atypical antipsychotic approved by the Food and Drug Administration (FDA) as an antipsychotic agent that blocks multiple neurotransmitters [72]. In recent studies, olanzapine has proved to effectively treat CINV in combination with classic triple antiemetic therapy [73]. However, the same study notes significant daytime somnolence as a side effect. These neuromodulators affect gut-brain interactions, which address chronic nausea symptoms unresponsive to conventional antiemetics [71].

Emerging antiemetic options such as cannabinoids, vitamin B6, ghrelin receptor antagonists, and neuromodulators are being researched as alternative antiemetic treatments. These treatments could offer better tolerability or relief when conventional therapies fail. Further studies are needed to understand their full use in clinical practice.

Discussion

The management of nausea and emesis is critical in improving patient outcomes across various clinical settings, from chemotherapy to postoperative care. Recent advancements in antiemetic therapies have led to a better understanding of effective interventions and their limitations. However, gaps remain in the literature, necessitating further research to optimize treatment strategies for diverse patient populations. This discussion synthesizes key findings from clinical studies, examines the strengths and weaknesses of current approaches, and identifies future research directions to address unmet needs in anti-emesis. The clinical studies reviewed in this paper highlight several promising antiemetic therapies. Notably, 5-HT3 receptor antagonists, such as ondansetron, have shown consistent efficacy in reducing nausea and vomiting across various settings, particularly CINV [39]. Other classes, including NK-1 receptor antagonists such as aprepitant, have demonstrated added benefits when combined with 5-HT3 antagonists, underscoring the utility of multi-mechanism approaches for managing refractory cases [28]. In addition to pharmacological treatments, recent studies have explored nontraditional therapies such as acupuncture, ginger, and cannabinoid compounds. These alternative approaches appear to offer supplementary benefits, particularly for patients who cannot tolerate standard antiemetic drugs or seek adjunctive therapies [63]. However, evidence remains limited, with small sample sizes and mixed results, indicating the need for further validation in larger, controlled trials [64].

The studies reviewed provide substantial evidence supporting multi-agent therapies as a superior approach to single-agent treatments in high-risk emetic scenarios. For example, the combination of a 5-HT3 antagonist, an NK-1 antagonist, and dexamethasone is now a standard regimen for high-risk CINV, significantly reducing nausea and vomiting rates compared to monotherapy [11]. This efficacy underscores the complementary mechanisms through which these drugs act on different receptors in the emetic pathway. Moreover, emerging therapies, including neurostimulation and certain herbal supplements, show potential in addressing nausea and vomiting in cases where pharmacotherapy alone is insufficient or contraindicated​ [71]. However, variability in outcomes suggests that individual responses to these therapies may depend on factors such as genetics, prior drug exposure, and the specific type of emetic trigger (e.g., opioid-induced versus motion sickness) [46]. The interpretation of these results suggests that while the pharmacological landscape for antiemetics is broadening, the personalization of treatment remains a key goal for future antiemetic strategies. Despite progress, several limitations persist within the current body of anti-emesis literature. Many research studies suffer from small sample sizes and limited follow-up, which can obscure long-term efficacy and safety profiles. For instance, although newer agents such as NK-1 antagonists show promise in clinical trials, there is limited real-world evidence on their long-term use in diverse populations, particularly among elderly patients or those with comorbid conditions [55]. Another significant gap lies in the underrepresentation of specific patient subgroups. Most antiemetic studies focus on chemotherapy-induced or postoperative nausea, often excluding patients with chronic conditions (e.g., gastrointestinal disorders or neurological diseases) who may experience nausea and vomiting but lack targeted treatment options. Furthermore, the efficacy of alternative therapies, such as cannabinoids and acupuncture, remains underexplored due to inconsistent methodologies and limited regulatory approval, highlighting the need for more rigorous clinical trials in these areas [32].

Addressing these gaps requires a multifaceted research approach. Firstly, larger, multicenter trials with extended follow-up periods are important to establish the long-term safety and efficacy of established and emerging antiemetic agents. Such studies should aim to include a broader range of patient demographics, including the elderly and those with coexisting health conditions, to understand the differential responses better and tailor therapies accordingly [58]. Secondly, personalized medicine approaches hold promise in optimizing antiemetic treatment. Research into genetic polymorphisms affecting antiemetic drug metabolism could inform more precise dosing and reduce adverse effects [45]. Similarly, exploring biomarkers that predict responsiveness to specific antiemetic classes may enable clinicians to select the most effective therapies for individual patients. Finally, alternative therapies warrant further investigation, especially given patients' growing interest in non-pharmacological options. Future research should prioritize large-scale, placebo-controlled studies on acupuncture, ginger, and cannabinoids to validate their efficacy and elucidate their mechanisms [31]. Additionally, examining the synergistic effects of combining pharmacological and non-pharmacological treatments could reveal new pathways for comprehensive nausea management [70].

In summary, while advances in anti-emesis research have improved patient outcomes, significant challenges remain. Though effective for many, current therapies are not universally successful across all patient populations and emetic triggers. Future research should focus on expanding trial inclusivity, exploring genetic and biomarker-guided treatment approaches, and rigorously evaluating alternative therapies. Through such efforts, it may be possible to achieve more individualized and effective antiemetic strategies, ultimately enhancing the quality of care for cases suffering from nausea and vomiting across diverse medical contexts (Tables 1, 2).

**Table 1:** Comparison of Pharmacodynamic and Pharmacokinetic Properties for Common Antiemetic Agents 5-HT, 5-hydroxytryptamine; GI, gastrointestinal; CNS, central nervous system; NK-1, neurokinin-1; CYP, cytochrome P450; IV, intravenous; IM, intramuscular

Drug	Mechanism of action (MOA)	Delivery and metabolism
Ondansetron	5-HT receptor antagonist in GI tract and CNS	IV/oral administration, metabolized by CYP450, and renal excretion
Metoclopramide	D2 receptor antagonist	IV/oral, hepatic metabolism, and renal excretion
Dexamethasone	Glucocorticoid with unclear antiemetic MOA	IV/oral, hepatic metabolism, and fecal/renal excretion
Aprepitant	NK-1 receptor antagonist	Oral, metabolized by CYP3A4, and fecal excretion
Promethazine	H1 receptor antagonist (CNS)	IV/oral/rectal, hepatic metabolism, and renal excretion
Scopolamine	Antimuscarinic (M1 in CNS)	Transdermal patch, hepatic metabolism, and renal excretion
Prochlorperazine	D2 receptor antagonist (CNS)	IM/oral/rectal, hepatic metabolism, and renal excretion
Diphenhydramine	H1 receptor antagonist (CNS)	IV/IM/oral, hepatic metabolism, and renal excretion

**Table 2:** Comparison of Side Effects and Contraindications of Common Antiemetic Agents GI, gastrointestinal; CNS, central nervous system; MAOi, monoamine oxidase inhibitor

Drug	Side effects	Contraindications
Ondansetron	Headache, constipation, dizziness, and serotonin syndrome	QT prolongation
Metoclopramide	Drowsiness, fatigue, and extrapyramidal symptoms	Parkinson's disease and GI obstruction
Dexamethasone	Insomnia, hyperglycemia, and mood changes	Systemic fungal infections and peptic ulcer disease
Aprepitant	Fatigue, hiccups, and dizziness	Known hypersensitivity
Promethazine	Sedation, dry mouth, blurred vision, and hypotension	Glaucoma and respiratory depression (children < 2 years)
Scopolamine	Dry mouth, drowsiness, and blurred vision	Glaucoma and myasthenia gravis
Prochlorperazine	Sedation, hypotension, and extrapyramidal symptoms	CNS depression, hypotension, and Parkinson's disease
Diphenhydramine	Drowsiness, dry mouth, urinary retention, and blurred vision	Newborns/breastfeeding, MAOi therapy, and glaucoma

Specific relevance

There are a few antiemetics that possess somewhat unique properties that may lend them special relevance in specific cases. For example, antihistamines and anticholinergics can grossly reduce the excitability of the vestibular system, which cuts off a motion sickness problem at the root [27]. Diphenhydramine has been used for decades and is trusted by patients, yet H2RAs have significantly fewer side effects as they do not cross the blood-brain barrier [52]. Scopolamine, an anticholinergic, carries the benefit of coming in a transdermal patch. This allows for easy at-home administration while simultaneously avoiding the GI tract in more sensitive patients. Similarly, for CINV, granisetron may be administered transdermally instead of for motion sickness [42]. In an emergency department setting, however, a quick IM injection may be preferred, in which case one might consider prochlorperazine, haloperidol, or metoclopramide, all D2 antagonists [39]. Lorazepam, or other benzodiazepines, could be strongly desirable in many types of anxiety-related emesis, as it palliates both psychological and physiological causes [32]. It should also be noted that there have been several multimodal therapies that have shown increased efficacy compared to monotherapy, especially in chemotherapy-induced nausea and vomiting (CINV) [11].

The standard alcohol prep pad that contains isopropyl alcohol can potentially treat nausea in the short term and has been used by health professionals for decades [74]. It is believed the noxious smell stimulates a sympathetic response, which overdrives parasympathetically mediated emesis.

Furthermore, a few drugs have significant negative considerations, which may indicate choosing an alternative option. First, it should be noted that nearly all the antiemetics addressed here undergo hepatic metabolism and renal excretion [44]. With this in mind, it is always important to reconsider conventional dosage in patients with liver or kidney failure. This is especially true of NK-1 and CYP3A4 inhibitors [4]. Even in healthy patients, this class can cause significant drug interactions. First-generation antihistamines can cause significant CNS effects, such as sedation, by crossing the blood-brain barrier. While H2RAs avoid these effects, cimetidine should be used with caution in men due to its anti-androgenic effects [53]. As with all glucocorticoids, dexamethasone should be used with great caution in suspect or confirmed diabetic patients [59]. Cannabinoids, though they have shown promise and have been FDA-approved to treat CINV, may have limited usefulness due to their psychoactive effects at these doses [63]. Finally, there is limited research concerning the side effects of droperidol at recommended antiemetic doses (0.625 mg to 1.25 mg) compared to higher doses (25 mg to 250 mg), which note significant QT prolongation. Further research in this field is needed to verify its safety.

Limitations

This review is somewhat limited in scope because of its qualitative reporting nature. The authors' goal was to compile a concise resource to lightly touch on the many options and considerations for treating emetic patients. For the sake of brevity, statistics from clinical trials and other minutiae have been omitted. Should the reader wish to consider in depth any drug mentioned here, the cited sources provide more than enough information, along with secondary resources, to satisfy a physician's need for numerical evidence.

## Conclusions

Drugs that act as antiemetics have a broad range across the medical field regarding mode of action and clinical relevance. This review intended to identify each class and consider the different applications they could fall into. With the above information in mind, it should be easier to make an informed decision when confronted with a difficult or refractory case of emesis.
